# Multimodal machine learning predicts type 2 respiratory failure in COPD exacerbations: a multicenter XGBoost model with clinical nomogram

**DOI:** 10.3389/fmed.2026.1806614

**Published:** 2026-07-15

**Authors:** Yunyu Liu, Yang Zhou, Yalian Zhang, Juntao Tan, Jun Gong

**Affiliations:** 1 Department of Medical Insurance, Affiliated Banan Hospital of Chongqing Medical University, Chongqing, China; 2 Medical Department, Affiliated Hospital of Nantong University, Jiangsu, China; 3 Department of Rehabilitation, Children’s Hospital of Chongqing Medical University, Chongqing, China; 4 College of Medical Informatics, Chongqing Medical University, Chongqing, China; 5 Department of Information Technology, People's Hospital of Chongqing Hechuan, Chongqing, China

**Keywords:** AECOPD, external validation, machine learning, nomogram, type 2 respiratory failure

## Abstract

**Background:**

Acute exacerbations of chronic obstructive pulmonary disease (AECOPD) frequently lead to life-threatening type 2 respiratory failure (T2RF). Existing predictive models rely on single biomarkers or linear methods and lack rigorous external validation. This study aimed to develop a multimodal machine learning framework to predict in-hospital T2RF risk with temporal–geographic external validation.

**Methods:**

We employed a two-source design. A development cohort of 6,954 AECOPD patients from a single tertiary hospital (2023–2025) was randomly divided into training (*n* = 4,867) and internal test (*n* = 2,087) sets. A temporal external validation cohort included 1,252 patients from seven hospitals (2016–2020). Eighteen admission predictors were evaluated. Missing values were imputed using missForest. Hybrid feature selection (LASSO plus XGBoost ranking) identified key variables. Six algorithms—logistic regression, SVM, random forest, GBDT, LightGBM, and XGBoost—were compared. Performance was assessed by AUROC, sensitivity, specificity, calibration, decision curve analysis, and SHAP values. A logistic nomogram was constructed.

**Results:**

In the internal test set, XGBoost achieved an AUROC of 0.660 (95% CI: 0.631–0.689). In the external validation set, XGBoost achieved an AUROC of 0.699 (95% CI: 0.661–0.738), with 45.9% sensitivity and 79.0% specificity. LightGBM performed comparably (AUROC 0.700). Seven predictors were selected: lymphocyte count, eosinophil count, COPD duration, RDW-CV, age, hypertension, and sex. SHAP analysis identified low lymphocyte count and long COPD duration as dominant risk drivers. The logistic nomogram achieved an external AUROC of 0.666.

**Conclusion:**

This externally validated framework enables early T2RF risk stratification at admission using routine blood counts and demographics. Future work should integrate dynamic monitoring and prospective multicenter validation.

## 1 Introduction

Chronic obstructive pulmonary disease (COPD), a leading global cause of morbidity and mortality, affects over 391 million individuals worldwide and is projected to become the third leading cause of death by 2030 (1, 2). Acute exacerbations of COPD (AECOPD), characterized by acute worsening of respiratory symptoms, are critical events that accelerate lung function decline, increase hospitalization rates, and contribute to a mortality rate of up to 8.3% during hospitalization (3, 4). Among the life-threatening complications of AECOPD, type 2 respiratory failure (T2RF), marked by hypercapnia (PaCO₂ ≥ 50 mmHg) and hypoxemia (PaO₂ < 60 mmHg), is particularly devastating, accounting for 24.5% of in-hospital mortality in AECOPD patients. This underscores the urgent need for early identification of high-risk patients to enable timely interventions, such as non-invasive ventilation or anti-inflammatory therapies, which have been shown to reduce mortality by 20% (5, 6).

Current predictive approaches for T2RF in AECOPD remain limited. Traditional models often rely on single biomarkers, such as C-reactive protein (CRP) or isolated blood gas parameters, which fail to capture the multifactorial pathophysiology of T2RF involving systemic inflammation, hypercoagulability, and ventilatory dysfunction (7–9). For instance, studies have highlighted the prognostic value of neutrophil-to-lymphocyte ratio (NLR), CRP, and systemic immune-inflammation index (SII) in assessing COPD severity, yet these markers are rarely integrated into comprehensive predictive frameworks (10, 11). Furthermore, conventional statistical methods, such as logistic regression, assume linear relationships between predictors and outcomes, neglecting complex interactions and temporal dynamics in clinical data (8, 12).

Recent advances in machine learning (ML) offer transformative potential for addressing these limitations (13). Techniques like LASSO regression and ensemble algorithms (e.g., XGBoost) enable robust feature selection and nonlinear modeling, improving predictive accuracy in high-dimensional datasets. For example, studies using random forests and support vector machines (SVM) have demonstrated moderate success in predicting postoperative respiratory failure, but their application to T2RF in AECOPD remains underexplored (14–16). Additionally, while nomograms derived from logistic regression provide clinically interpretable tools for resource-limited settings, their performance often lags behind ML models in handling class imbalance and capturing intricate predictor-outcome associations (17).

This study was designed as a two-source temporal–geographic validation framework to maximize generalizability and bedside utility. A contemporary development cohort of 6,954 AECOPD patients (2023–2025) from a single tertiary hospital was used for model training and internal testing, while a historically distinct multicenter cohort of 1,252 patients (2016–2020) from seven hospitals served as an independent temporal external validation set. Recognizing that spirometry is frequently unavailable during acute exacerbations, pulmonary ventilatory function (PVF) was deliberately excluded, restricting predictors to routinely available admission blood counts and demographic data. Furthermore, the conventional 48-h post-admission exclusion window was eliminated, enabling prediction of any in-hospital T2RF event from admission parameters alone.

To enhance generalizability and bedside utility, this study developed a multimodal machine learning framework for predicting in-hospital type II respiratory failure (T2RF) among patients with acute exacerbation of chronic obstructive pulmonary disease (AECOPD). Recognizing that spirometry is frequently unavailable during acute exacerbations, we deliberately excluded pulmonary ventilatory function and restricted predictors to routinely available admission blood counts and demographic data, while eliminating the conventional 48-h post-admission exclusion window to enable prediction of any in-hospital T2RF event from admission parameters alone. Leveraging a two-source temporal–geographic validation framework—comprising a contemporary development cohort of 6,954 patients (2023–2025) from a single tertiary hospital and a historically distinct multicenter external validation cohort of 1,252 patients (2016–2020) from seven hospitals—we employed a hybrid LASSO–XGBoost feature selection strategy to integrate inflammatory markers (e.g., lymphocyte and eosinophil counts), coagulation indicators (e.g., D-dimer), and clinical parameters (e.g., COPD duration, age). This approach addresses the limitations of single-biomarker studies and provides a practical nomogram for frontline clinicians, aligning with precision medicine principles in COPD management.

## 2 Materials and methods

### 2.1 Study design and participants

This study used two distinct retrospective data sources. Source 1 (Development cohort) comprised hospitalized patients with AECOPD from a tertiary academic medical center in Chongqing, China, between January 1, 2023, and December 31, 2025. Source 2 (External validation cohort) comprised hospitalized AECOPD patients from seven affiliated medical institutions of Chongqing Medical University between January 1, 2016, and December 31, 2020. The inclusion criteria were as follows: (1) age ≥40 years; (2) a primary diagnosis of AECOPD according to the International Classification of Diseases, 10th Edition (ICD-10: J44.1 and J44.0); and (3) admission laboratory tests available within 24 h of hospitalization. The T2RF diagnostic criteria were the partial pressure of oxygen in arterial blood (PaO2) < 60 mmHg and the partial pressure of carbon dioxide in arterial blood (PaCO2) > 50 mmHg under ambient air (18). The exclusion criteria were as follows (19): (1) Patients with active pulmonary tuberculosis, pulmonary fibrosis, pulmonary embolism, pneumothorax, and lung cancer. (2) Patients with immunodeficiency-related diseases. (3) Patients with anemia or hematological diseases of various causes. (4) Patients with severe hepatic and renal insufficiency. (5) Patients admitted for respiratory failure. Owing to the feature of retrospective study, patients’ informed consent is not required. The study protocol was approved by the Ethics Committee of Chongqing Medical University and the Ethics Committee of the tertiary development center, conducted in accordance with the Declaration of Helsinki.

### 2.2 Candidate predictors

Predictors satisfying the following criteria were included: (1) obtained within 24 h of admission; (2) missing rate <30%; and (3) routinely available in general hospitals without specialized equipment. The final 18 predictors were: age, sex, smoking status, hypertension, diabetes, coronary heart disease (CHD), COPD duration, neutrophil count (NEUT#), neutrophil percentage (NEUT%), eosinophil count (EO#), eosinophil percentage (EO%), lymphocyte count (LYMPH#), lymphocyte percentage (LYMPH%), neutrophil-to-lymphocyte ratio (NLR), white blood cell count (WBC), red cell distribution width–coefficient of variation (RDW-CV), procalcitonin (PCT), and D-dimer (D-D).

Missing-value handling. For predictors with missing rates <30%, missing values were imputed using the missForest package (version 1.5) in R (version 4.3.1). missForest uses a random-forest algorithm that iteratively predicts missing values from observed data, preserving mixed-type distributions and inter-variable relationships. To prevent data leakage, missForest was fitted exclusively on the training set (*n* = 4,867); the trained imputation model was then applied to the internal test and external validation sets. The missing rates in the development cohort were: PCT 27.7%, D-D 8.8%, COPD duration 14.3%, and all other predictors <5% (Supplementary Table S1).

### 2.3 Statistical analysis and machine learning pipeline

Machine learning model development and evaluation were implemented in Python (version 3.10), using scikit-learn (GBDT and preprocessing), xgboost, lightgbm, and SHAP (model explanation). All other statistical analyses were performed in R (version 4.3.1), including LASSO (glmnet), missing data imputation (missForest), logistic regression and nomogram construction (rms), SVM (e1071), and random forest (randomForest). Categorical variables were summarized as frequencies with percentages, and continuous variables as medians with interquartile ranges (IQR) or means with standard deviations, as appropriate. Between-group comparisons for continuous variables used the Mann–Whitney U test or independent *t*-test, and categorical variables were compared with the chi-square test.

The development cohort (*n* = 6,954) was randomly divided into a training set (70%, *n* = 4,867) for model derivation and an internal test set (30%, *n* = 2,087) for preliminary validation, employing stratified randomization to preserve the case–control ratio. The independent temporal–geographic external validation cohort (*n* = 1,252) was retained to assess generalizability. To prevent data leakage, the missForest imputation model was fitted exclusively on the training set and subsequently applied to both the internal test and external validation sets.

Feature selection followed a hybrid two-step procedure. First, univariate analysis screened candidate predictors with *p* < 0.05. Second, LASSO logistic regression with 10-fold cross-validation was performed on the training data to shrink redundant coefficients toward zero. The optimal regularization parameter (*λ* = 0.0021) was selected using the minimum deviance plus one standard error (1-SE) criterion, yielding seven non-zero predictors. The stability of these selected features was further confirmed by gain-based importance ranking from a preliminary XGBoost model.

Six supervised learning algorithms were trained and compared under identical conditions: logistic regression (LR), random forest (RF), gradient boosting decision tree (GBDT), LightGBM, XGBoost, and decision tree (DT). For each tree-based ensemble, hyperparameters were optimized via randomized search with 5-fold cross-validation on the training set, maximizing the area under the receiver operating characteristic curve (AUROC). All models were fitted using the same seven selected features to ensure a fair comparison.

Discrimination was evaluated by AUROC with 95% confidence intervals (CIs) estimated by 1,000 bootstrap replications, together with sensitivity, specificity, Youden index, and F1-score. Calibration was graphically examined with calibration curves and quantified by the Brier score. Clinical utility was appraised with decision curve analysis (DCA) across the full range of threshold probabilities. Model interpretability was enhanced by SHAP (SHapley Additive exPlanations) values computed from the final XGBoost model to delineate the magnitude and direction of each predictor’s contribution at both the population and individual levels. Additionally, a logistic regression-based nomogram incorporating the seven selected variables was constructed to provide a clinically actionable bedside tool. All statistical tests were two-sided, and *p* < 0.05 was considered statistically significant.

## 3 Results

### 3.1 Study population and baseline characteristics

**Figure 1:**
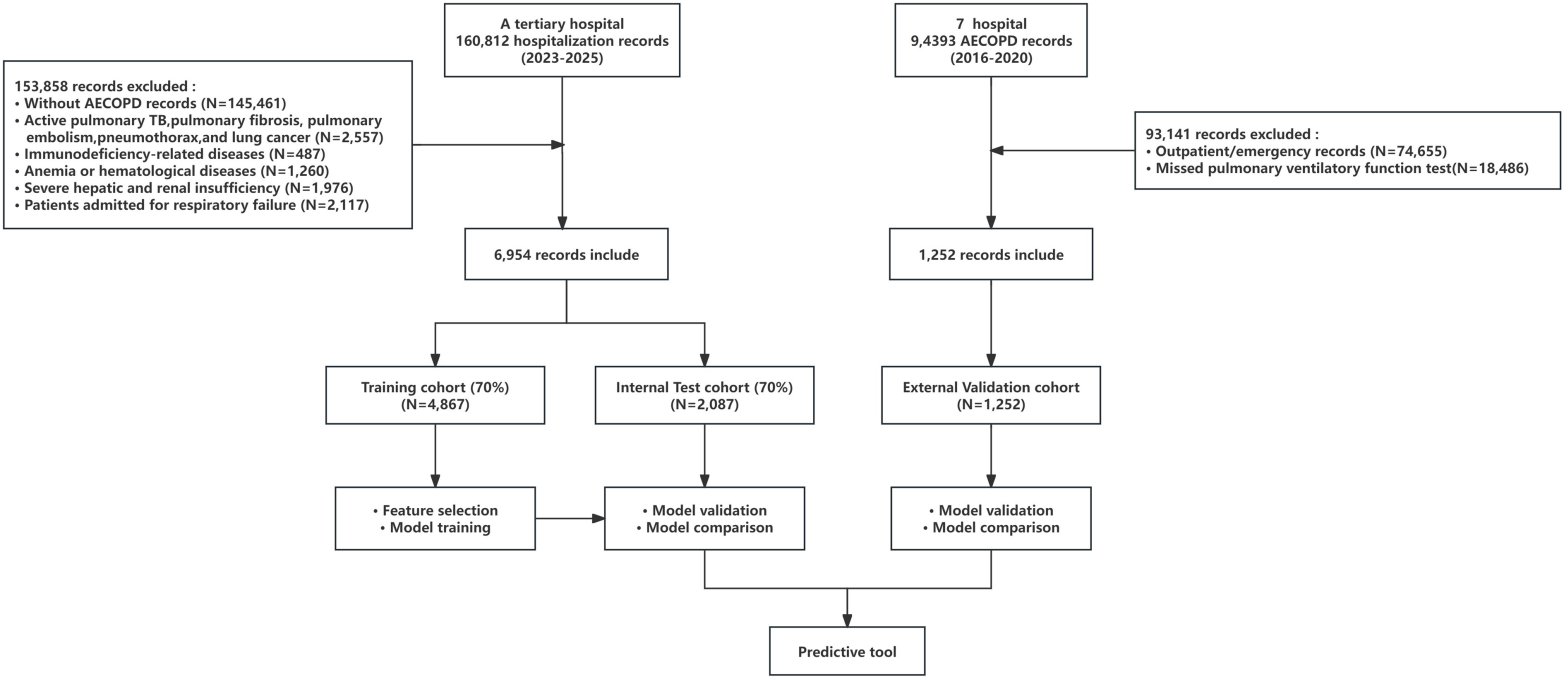
Study flowchart.

The study flowchart is presented in Figure 1. Of the 160,812 hospitalization records screened at the tertiary development center between 2023 and 2025, 153,858 were excluded according to the predefined criteria, yielding 6,954 eligible AECOPD patients. These were randomly partitioned into the training set (*n* = 4,867) and the internal test set (*n* = 2,087) using stratified randomization to preserve the outcome distribution. The independent temporal–geographic external validation cohort comprised 1,252 patients from seven hospitals (2016–2020) after excluding 93,141 ineligible records.

Baseline characteristics stratified by T2RF status are summarized in Table 1. Patients who developed T2RF were significantly older, more likely to be male, and had a longer COPD duration than those without T2RF. Laboratory analyses revealed that the T2RF group exhibited lower lymphocyte counts and percentages, lower eosinophil counts and percentages, higher neutrophil percentages, an elevated neutrophil-to-lymphocyte ratio (NLR), higher procalcitonin (PCT), higher D-dimer, and higher red cell distribution width–coefficient of variation (RDW-CV; all *p* < 0.05). Significant differences were also observed in the prevalence of hypertension, diabetes, and coronary heart disease between the two groups.

**Table 1:** Characteristics of AECOPD patients complicated with T2RF or not.

Predictors	Training (3,323)		Internal test (1,425)		External validation (1,252)		*p*
	Without T2RF (2,683)	With T2RF (640)	Without T2RF (1,151)	With T2RF (274)	Without T2RF (1,010)	With T2RF (242)	
Age	76.00 [69.00, 81.00]	73.00 [67.00, 80.00]	75.00 [70.00, 81.00]	74.00 [68.00, 80.00]	75.00 [69.00, 81.00]	75.00 [68.00, 81.00]	<0.001
Sex							0.001
Female	797 (20.2)	227 (24.8)	322 (19.0)	84 (21.4)	243 (24.1)	44 (18.2)	
Male	3,156 (79.8)	687 (75.2)	1,373 (81.0)	308 (78.6)	767 (75.9)	198 (81.8)	
Smoke							<0.001
Yes	1,550 (39.2)	360 (39.4)	661 (39.0)	142 (36.2)	562 (55.6)	116 (47.9)	
No	2,403 (60.8)	554 (60.6)	1,034 (61.0)	250 (63.8)	448 (44.4)	126 (52.1)	
Hypertension							<0.001
Yes	2,422 (61.3)	638 (69.8)	1,023 (60.4)	257 (65.6)	762 (75.4)	177 (73.1)	
No	1,531 (38.7)	276 (30.2)	672 (39.6)	135 (34.4)	248 (24.6)	65 (26.9)	
Diabetes							<0.001
Yes	3,341 (84.5)	788 (86.2)	1,449 (85.5)	345 (88.0)	945 (93.6)	219 (90.5)	
No	612 (15.5)	126 (13.8)	246 (14.5)	47 (12.0)	65 (6.4)	23 (9.5)	
CHD							<0.001
Yes	2,604 (65.9)	632 (69.1)	1,101 (65.0)	260 (66.3)	914 (90.5)	220 (90.9)	
No	1,349 (34.1)	282 (30.9)	594 (35.0)	132 (33.7)	96 (9.5)	22 (9.1)	
COPD duration	10.00 [5.00, 14.00]	10.00 [9.00, 20.00]	10.00 [5.00, 11.00]	10.00 [10.00, 20.00]	10.00 [5.00, 10.00]	10.00 [10.00, 20.00]	<0.001
PCT	0.12 [0.05, 0.32]	0.13 [0.02, 0.36]	0.12 [0.04, 0.12]	0.12 [0.06, 0.16]	0.10 [0.05, 0.18]	0.08 [0.05, 0.14]	0.001
DD	0.35 [0.20, 0.62]	0.39 [0.21, 0.74]	0.35 [0.20, 0.55]	0.35 [0.23, 0.76]	0.59 [0.40, 0.71]	0.40 [0.20, 0.70]	<0.001
NEUT# [×10^9^/L]	5.97 [4.18, 8.38]	6.50 [4.49, 8.93]	6.07 [4.20, 8.27]	6.31 [4.61, 8.77]	5.03 [3.62, 7.00]	6.03 [4.36, 8.04]	<0.001
NEUT% [%]	77.90 [69.60, 84.60]	81.80 [74.70, 87.60]	78.00 [69.50, 84.40]	80.75 [75.27, 87.50]	72.00 [63.81, 79.80]	77.05 [68.23, 85.20]	<0.001
EO# [×10^9^/L]	0.08 [0.02, 0.17]	0.05 [0.01, 0.14]	0.08 [0.02, 0.18]	0.06 [0.01, 0.15]	0.11 [0.04, 0.19]	0.08 [0.02, 0.17]	<0.001
EO% [%]	1.10 [0.20, 2.50]	0.60 [0.10, 1.80]	1.00 [0.30, 2.50]	0.80 [0.10, 2.12]	1.60 [0.50, 3.07]	1.10 [0.26, 2.28]	<0.001
LYMPH# [×10^9^/L]	0.96 [0.66, 1.35]	0.74 [0.50, 1.05]	0.92 [0.65, 1.33]	0.72 [0.47, 1.09]	1.24 [0.92, 1.64]	0.99 [0.68, 1.51]	<0.001
LYMPH% [%]	12.60 [7.90, 19.40]	9.60 [5.70, 14.67]	12.10 [7.65, 19.20]	10.20 [5.47, 14.43]	17.94 [12.00, 25.00]	13.60 [7.80, 20.32]	<0.001
WBC [×10^9^/L]	7.80 [5.90, 10.20]	8.15 [5.80, 10.60]	7.90 [5.95, 10.10]	7.90 [6.20, 10.33]	7.13 [5.61, 9.18]	7.94 [6.23, 10.17]	<0.001
RDW-CV [%]	14.00 [13.00, 14.04]	14.00 [13.00, 15.00]	14.00 [13.00, 14.00]	14.00 [13.00, 15.00]	13.50 [12.90, 14.00]	13.70 [13.12, 14.50]	<0.001
NLR	6.19 [3.58, 10.80]	8.56 [5.06, 15.57]	6.39 [3.59, 11.03]	7.90 [5.23, 16.07]	4.00 [2.57, 6.65]	5.78 [3.42, 10.62]	<0.001

### 3.2 Feature selection

**Figure 2:**
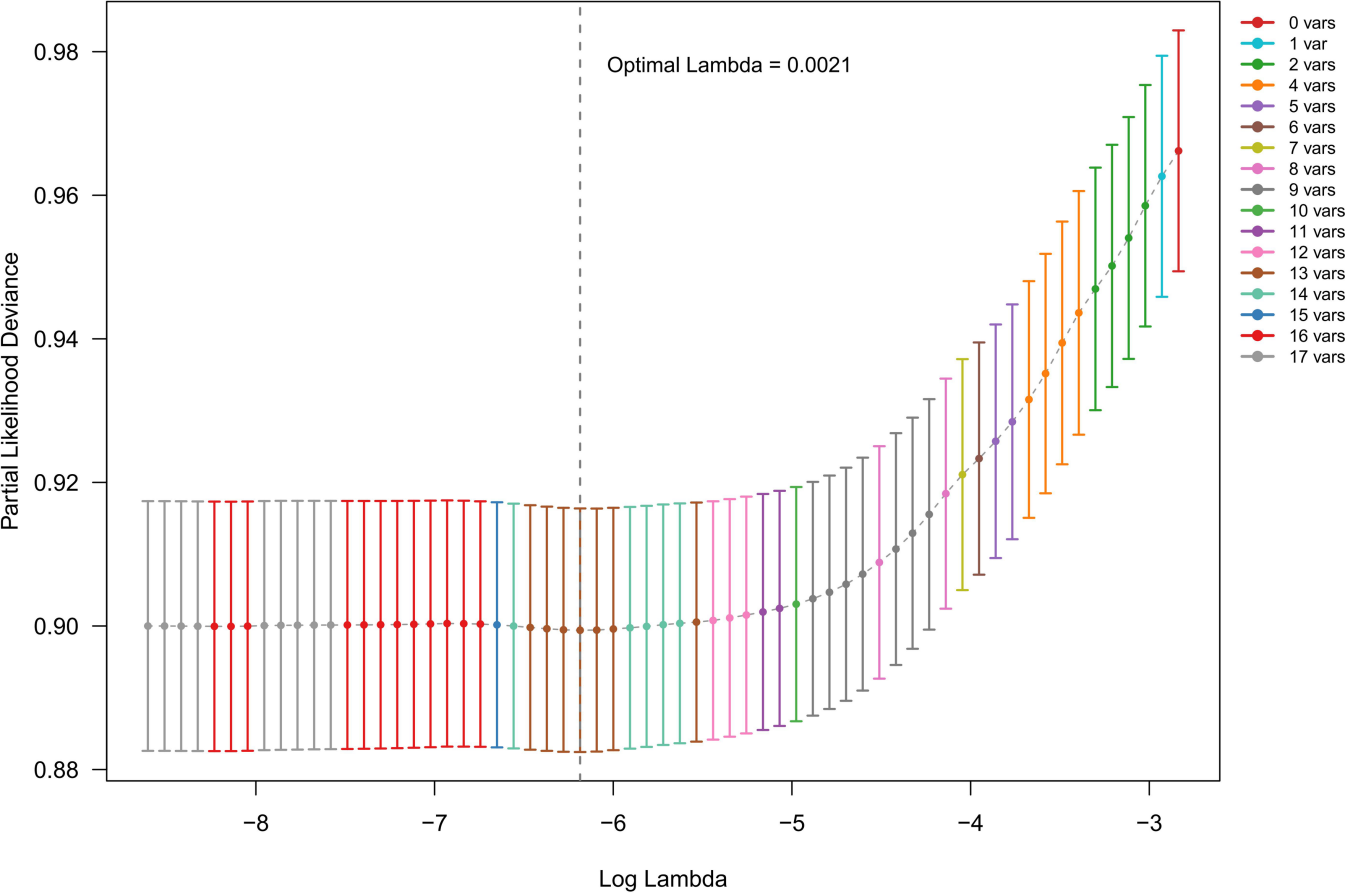
LASSO cross-validation curve. The optimal lambda (0.0021) was selected by the minimum deviance +1SE criterion, retaining seven predictors.

Of the 18 candidate admission predictors, those with *p* < 0.05 on univariate analysis were subsequently entered into LASSO logistic regression with 10-fold cross-validation. The optimal regularization parameter (*λ* = 0.0021) was determined by the minimum deviance plus one standard error criterion (Figure 2). This procedure retained seven predictors with non-zero coefficients: lymphocyte count (LYMPH#), eosinophil count (EO#), COPD duration, RDW-CV, age, hypertension, and sex. The robustness of these selected features was corroborated by gain-based importance ranking from a preliminary XGBoost model (Supplementary Table S2).

### 3.3 Model discrimination and comparison

**Figure 3:**
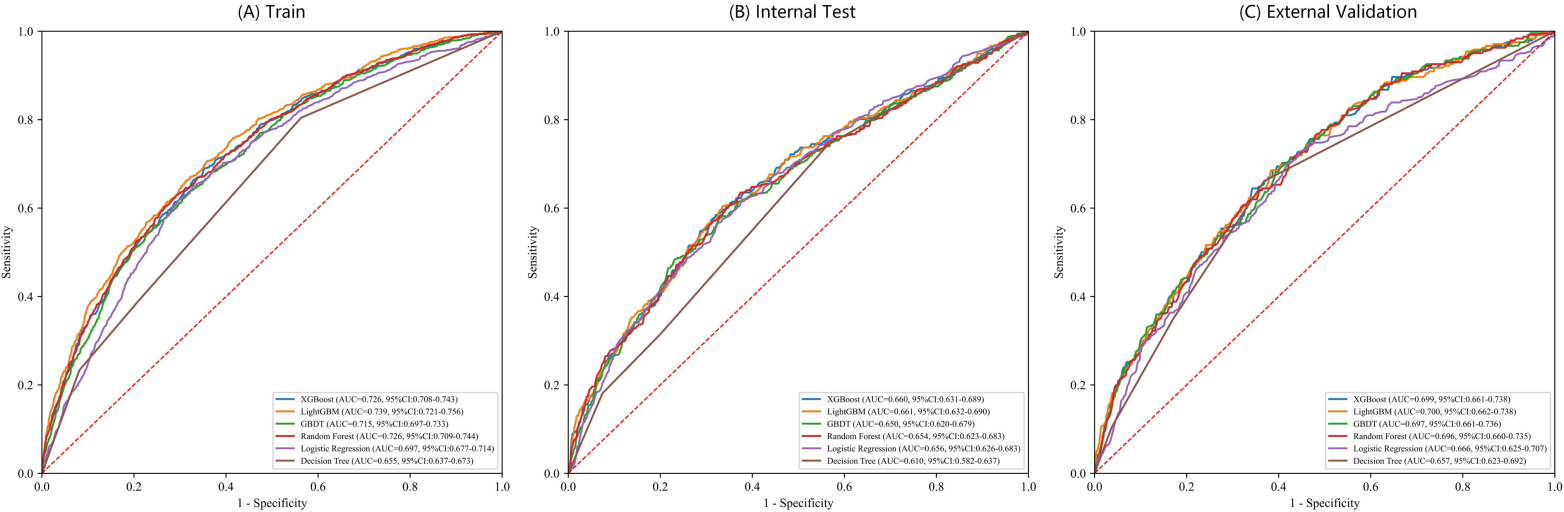
ROC curves for the six models across **(A)** training, **(B)** internal test, and **(C)** external validation cohorts.

Six algorithms—logistic regression, decision tree, random forest, GBDT, LightGBM, and XGBoost—were trained on the same seven-predictor set and evaluated across three cohorts. In the training set, LightGBM attained the highest AUROC (0.739, 95% CI: 0.721–0.756), closely followed by XGBoost and random forest (AUROC 0.726 each; Figure 3A and Table 2).

**Table 2:** Performance evaluation of six models in the internal test cohort (*n* = 2,087) and the external validation cohort (*n* = 1,252).

Model	Dataset	AUC (95%CI)	Sensitivity	Specificity	Youden_Index	F1_Score
XGBoost	Train	0.726 (0.708, 0.743)	0.672	0.653	0.325	0.424
	Internal test	0.660 (0.631, 0.689)	0.617	0.637	0.255	0.388
	External validation	0.699 (0.661, 0.738)	0.459	0.790	0.249	0.393
LightGBM	Train	0.739 (0.721, 0.756)	0.688	0.652	0.340	0.431
	Internal test	0.661 (0.632, 0.690)	0.615	0.622	0.237	0.378
	External validation	0.700 (0.662, 0.738)	0.471	0.782	0.253	0.396
GBDT	Train	0.715 (0.697, 0.733)	0.671	0.637	0.307	0.414
	Internal test	0.650 (0.620, 0.679)	0.615	0.629	0.244	0.382
	External validation	0.697 (0.661, 0.736)	0.442	0.809	0.251	0.395
Random forest	Train	0.726 (0.709, 0.744)	0.672	0.650	0.321	0.422
	Internal test	0.654 (0.623, 0.684)	0.610	0.641	0.251	0.386
	External validation	0.696 (0.660, 0.735)	0.434	0.803	0.237	0.385
Logistic regression	Train	0.697 (0.677, 0.714)	0.654	0.663	0.318	0.421
	Internal test	0.656 (0.626, 0.683)	0.589	0.647	0.236	0.378
	External validation	0.666 (0.625, 0.707)	0.455	0.786	0.241	0.387
Decision tree	Train	0.655 (0.637, 0.673)	0.804	0.437	0.241	0.379
	Internal test	0.610 (0.582, 0.637)	0.742	0.437	0.179	0.355
	External validation	0.657 (0.623, 0.692)	0.661	0.632	0.293	0.413

**Figure 4:**
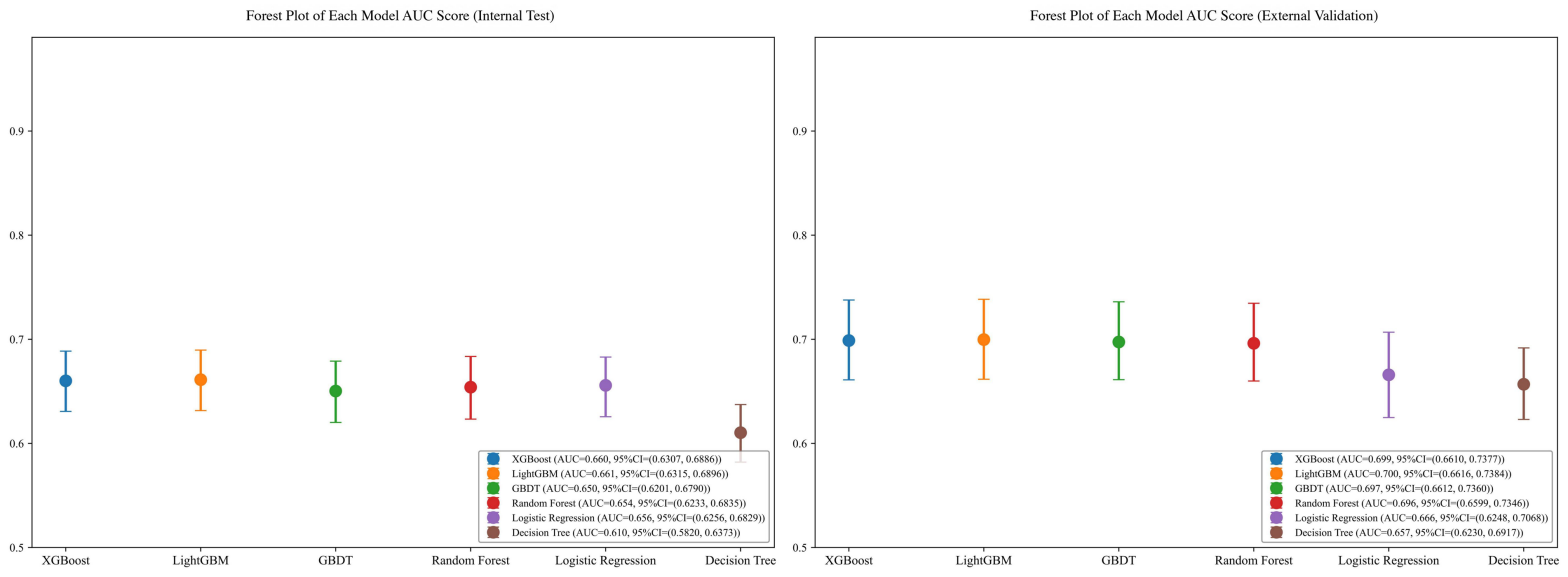
Forest plot of AUROC distributions for each model in the internal test (left) and external validation (right) sets.

In the internal test set, all tree-based ensembles demonstrated comparable discrimination, with LightGBM achieving an AUROC of 0.661 (95% CI: 0.632–0.690), XGBoost 0.660 (95% CI: 0.631–0.689), and logistic regression 0.656 (95% CI: 0.626–0.683; Figure 3B, Figure 4, and Table 2). The decision tree performed poorest (AUROC 0.610).

External validation in the temporally distinct multicenter cohort revealed superior generalizability for the gradient-boosting frameworks. LightGBM achieved an AUROC of 0.700 (95% CI: 0.662–0.738), XGBoost 0.699 (95% CI: 0.661–0.738), GBDT 0.697 (95% CI: 0.661–0.736), and random forest 0.696 (95% CI: 0.660–0.735), all substantially outperforming logistic regression (AUROC 0.666, 95% CI: 0.625–0.707) and decision tree (AUROC 0.657, 95% CI: 0.623–0.692; Figure 3C and Table 2). Notably, XGBoost exhibited a sensitivity of 45.9% and a specificity of 79.0% in this external cohort, whereas LightGBM showed comparable metrics (sensitivity 47.1%, specificity 78.2%). Forest plots of AUROC distributions confirmed overlapping but consistently higher point estimates for the boosting algorithms in both the internal and external sets (Figure 4). For clinical accessibility, a logistic regression-based nomogram incorporating the seven selected variables was constructed, which achieved an external AUROC of 0.666 (95% CI: 0.625–0.707), with a sensitivity of 45.5% and a specificity of 78.6%.

### 3.4 Calibration and clinical utility

**Figure 5:**
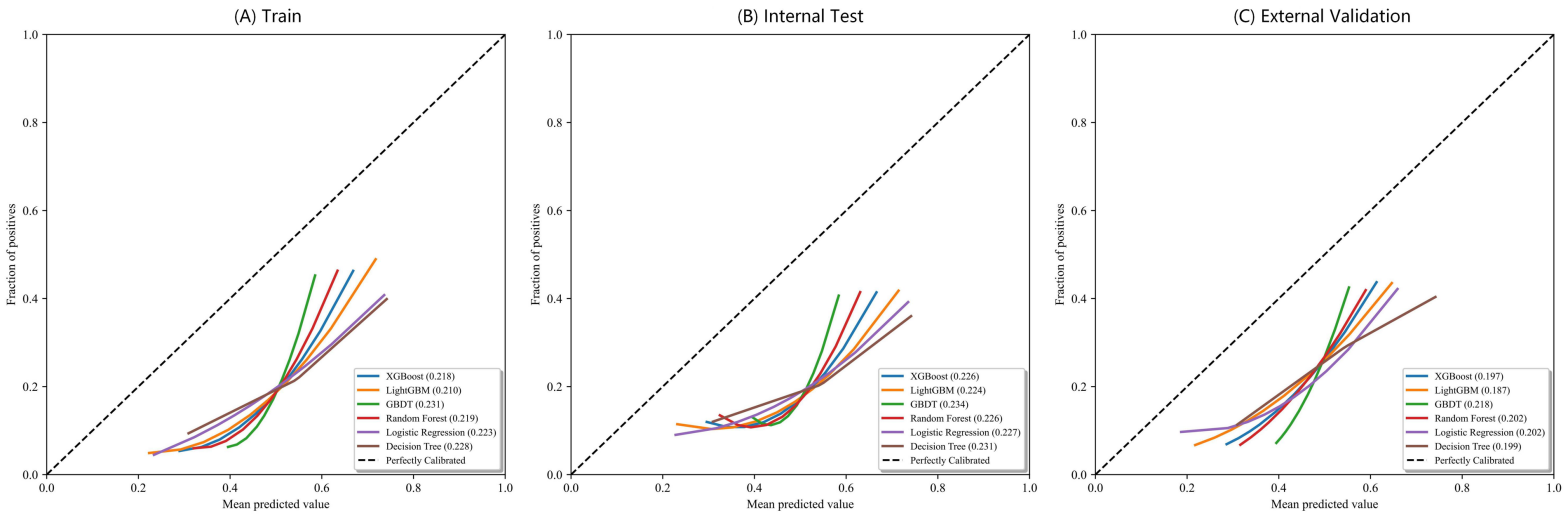
Calibration curves for all six models across **(A)** training, **(B)** internal test, and **(C)** external validation. Numbers in parentheses indicate Brier scores.

Calibration curves were used to assess the agreement between predicted probabilities and observed outcomes (Figure 5). In the training set, all models demonstrated acceptable calibration, with Brier scores ranging from 0.210 (LightGBM) to 0.231 (GBDT). Calibration remained stable in the internal test set (Brier scores 0.224–0.234) and improved slightly in the external validation set, where LightGBM recorded the lowest Brier score (0.187), followed by XGBoost (0.197).

**Figure 6:**
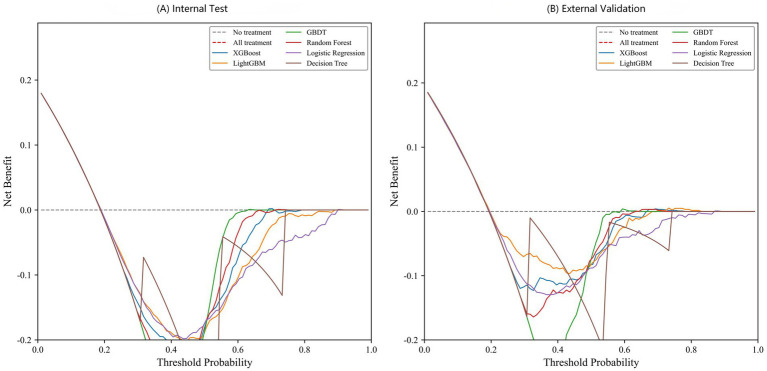
Decision curve analysis for **(A)** internal test and **(B)** external validation cohorts.

Decision curve analysis was conducted to evaluate the net clinical benefit across threshold probabilities (Figure 6). In both the internal test and external validation cohorts, XGBoost, LightGBM, GBDT, and random forest provided positive net benefit over the treat-all and treat-none strategies across a broad range of clinically relevant thresholds (approximately 0.5 to 0.8), whereas logistic regression and decision tree offered limited incremental value.

### 3.5 Model interpretability

**Figure 7:**
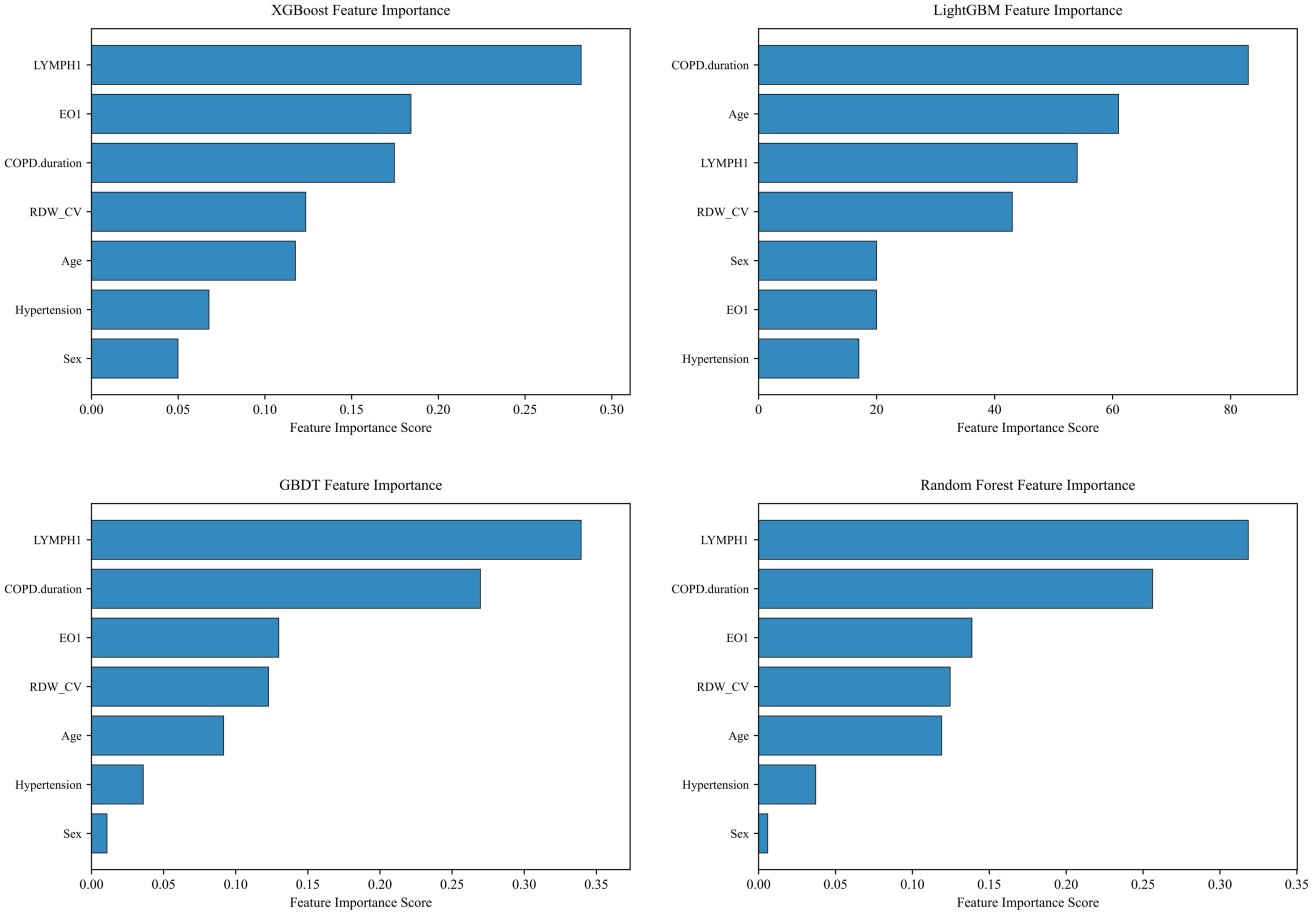
Feature importance rankings for XGBoost, LightGBM, GBDT, and random forest.

Feature importance rankings derived from the tree-based models are shown in Figure 7. Lymphocyte count and COPD duration consistently ranked as the top two predictors across XGBoost, GBDT, and random forest. LightGBM additionally emphasized age and RDW-CV.

**Figure 8:**
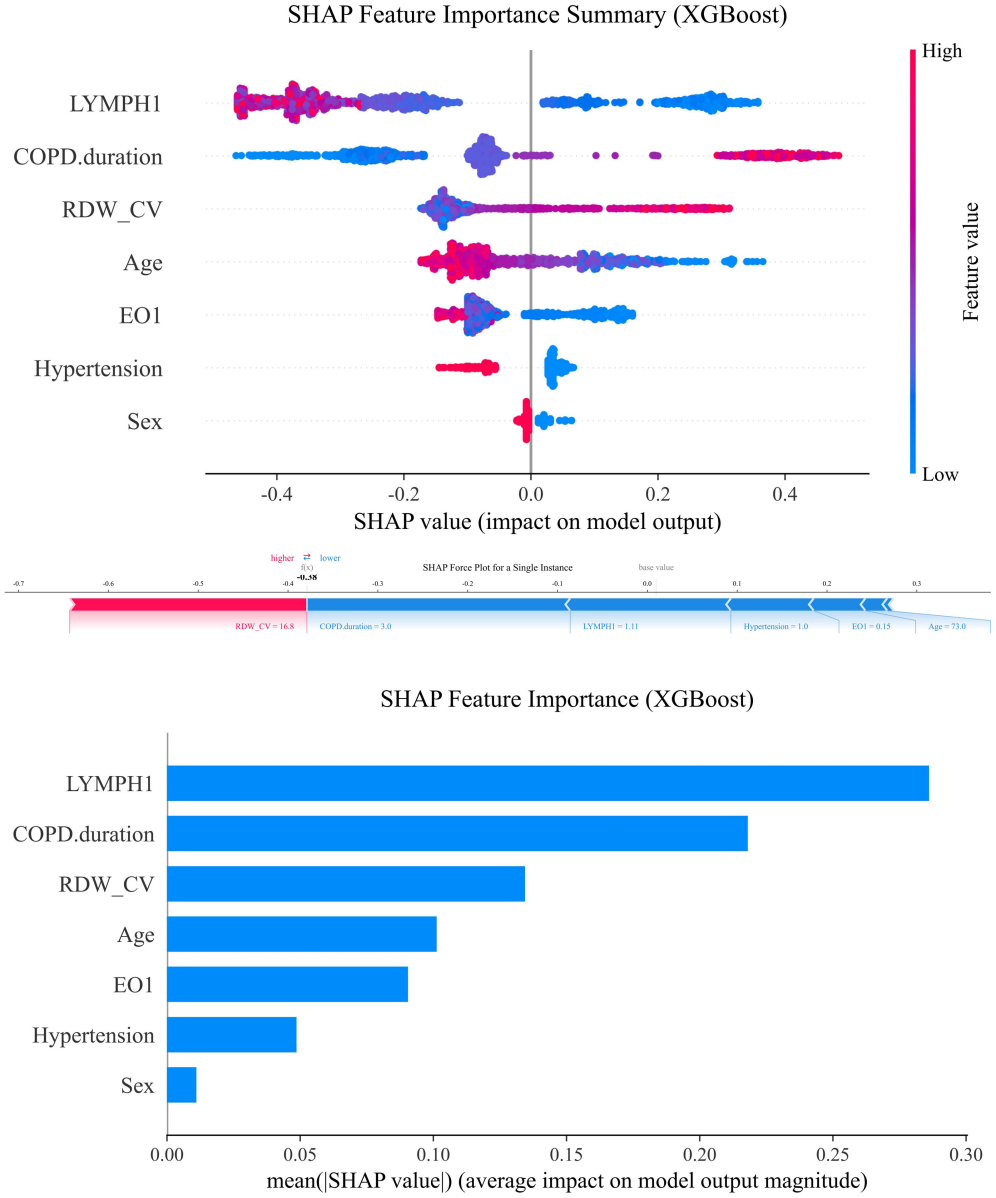
SHAP summary plot, force plot, and mean SHAP bar plot for XGBoost in the internal test cohort.

**Figure 9:**
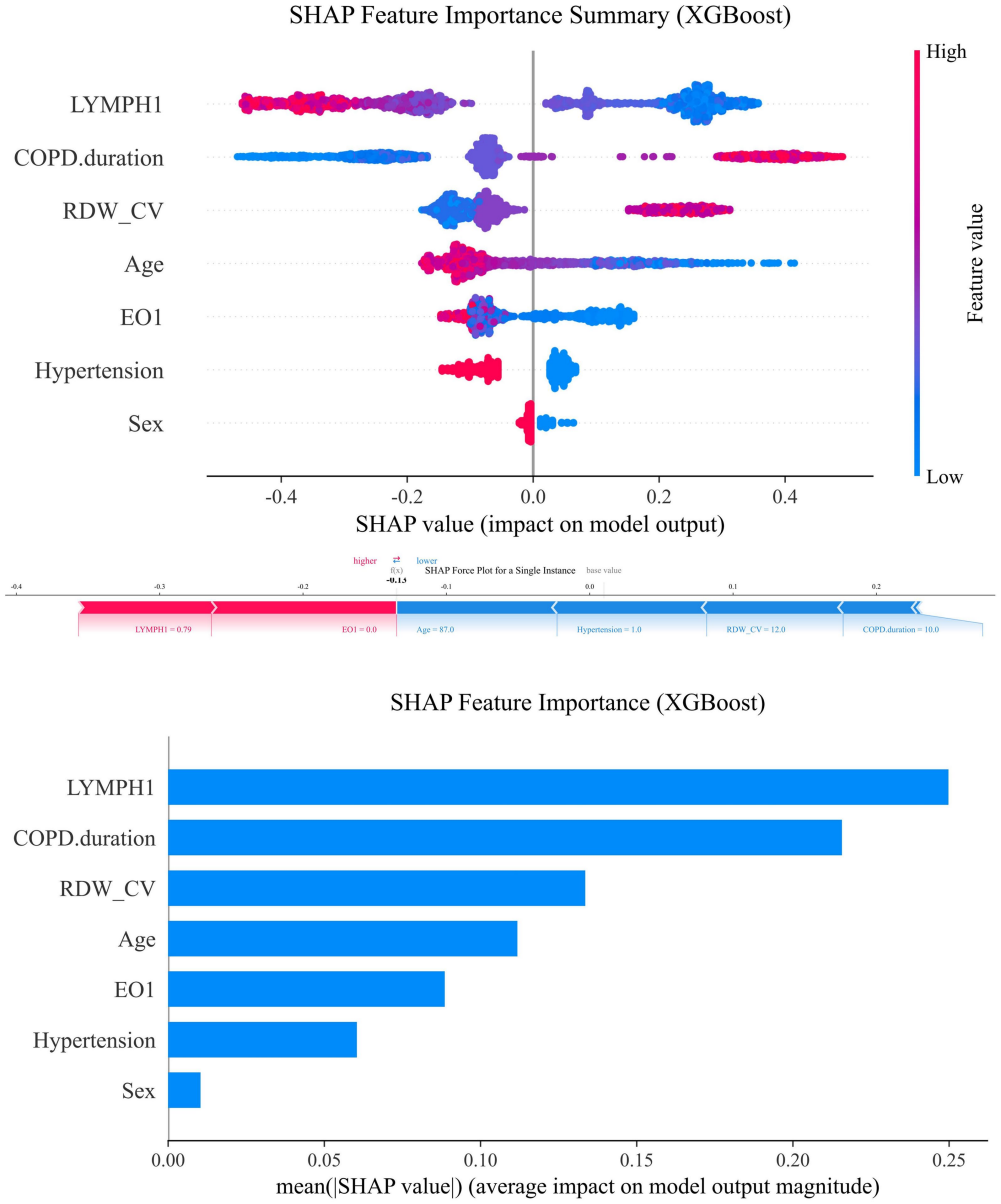
SHAP summary plot, force plot, and mean SHAP bar plot for XGBoost in the external validation cohort.

SHAP analysis for the final XGBoost model elucidated the directionality and magnitude of each feature’s contribution (Figures 8, 9). A low lymphocyte count emerged as the dominant risk driver, with lower values shifting predictions toward higher T2RF probability. Longer COPD duration, advanced age, higher RDW-CV, and lower eosinophil count also increased risk. Hypertension and male sex contributed modestly but consistently. SHAP force plots further illustrated how individualized predictions were derived from the interplay of these factors in representative cases.

## 4 Discussion

In this study, we developed and externally validated a multimodal machine learning framework for the early prediction of in-hospital type 2 respiratory failure (T2RF) among patients with acute exacerbation of chronic obstructive pulmonary disease (AECOPD). Through a hybrid feature selection strategy combining univariate analysis with LASSO regression, we identified seven admission predictors: lymphocyte count, eosinophil count, COPD duration, red cell distribution width–coefficient of variation (RDW-CV), age, hypertension, and sex. The optimized XGBoost model achieved an area under the receiver operating characteristic curve (AUROC) of 0.660 (95% CI: 0.631–0.689) in the internal test set and 0.699 (95% CI: 0.661–0.738) in the temporal–geographic external validation set, with 45.9% sensitivity and 79.0% specificity. LightGBM demonstrated comparable discriminative performance (external AUROC 0.700). SHAP analysis revealed that low lymphocyte count and prolonged COPD duration were the dominant risk drivers. Furthermore, a logistic regression-derived nomogram provided a clinically interpretable bedside tool with an external AUROC of 0.666. These findings suggest that our framework enables early T2RF risk stratification at admission using only routine blood counts and demographic data.

Current studies on T2RF prediction in AECOPD predominantly rely on single biomarkers, such as isolated assessments of inflammatory markers (e.g., C-reactive protein) or blood gas parameters (e.g., PaCO₂) (20, 21). While these markers provide partial insights, their predictive power is inherently limited by the multifactorial pathophysiology of T2RF, which involves systemic inflammation, immune dysregulation, and progressive ventilatory dysfunction (22, 23). Our study advances the field by integrating seven routine admission variables—encompassing immune cell counts (lymphocytes, eosinophils), erythrocyte indices (RDW-CV), and clinical demographics (COPD duration, age, hypertension, sex)—into a unified predictive framework. This approach captures both acute inflammatory status and chronic disease burden, overcoming the oversimplification of single-parameter models (24, 25). The superior discriminative capacity of our ensemble models (external AUROC: 0.699–0.700) compared with conventional logistic regression (AUROC: 0.666) underscores the value of multimodal integration in capturing complex predictor–outcome associations.

Traditional predictive models for T2RF often employ univariate screening or stepwise regression, which may overlook complex interactions among variables or suffer from overfitting in high-dimensional datasets (26, 27). In contrast, our hybrid feature selection strategy—combining univariate filtering with LASSO regularization—ensures rigorous variable screening while preserving critical predictors through L1 shrinkage. Subsequent application of XGBoost and LightGBM, gradient-boosted ensemble algorithms, further optimizes generalizability by handling non-linear relationships and class imbalance. This methodological synergy contrasts sharply with conventional logistic regression, which assumes linearity and independence of predictors (28–30). While the logistic regression nomogram in our study achieved an acceptable external AUROC of 0.666, the tree-based ensemble models consistently outperformed it across both internal and external evaluations, highlighting the advantage of machine learning in capturing intricate, non-additive effects (31–32). The XGBoost model’s robustness across temporally and geographically distinct cohorts likely stems from its ability to assign adaptive weights to misclassified samples and its built-in regularization against overfitting (33, 34).

The proposed predictive framework enables clinicians to identify high-risk AECOPD patients for in-hospital T2RF at admission, a critical window for initiating targeted interventions. Current clinical practices often rely on delayed assessments of respiratory failure, contributing to the persistently high mortality burden associated with T2RF in AECOPD (4, 5). By integrating lymphocyte count, eosinophil count, and RDW-CV—biomarkers reflective of systemic immune status and chronic inflammatory burden—the model facilitates early escalation of therapies such as non-invasive ventilation or anti-inflammatory regimens (35, 36). For instance, timely non-invasive ventilation has been shown to reduce intubation rates and mortality in hypercapnic respiratory failure (36). Our model’s moderate-to-high specificity (79.0% for XGBoost, 78.2% for LightGBM) minimizes unnecessary interventions in low-risk patients, while its sensitivity ensures clinically acceptable detection of high-risk cases.

The logistic regression-derived nomogram provides a user-friendly, point-of-care tool that requires no specialized computational resources, making it particularly valuable for primary care facilities in low-resource regions (37, 38). Clinicians can calculate individualized T2RF risk scores using routinely available admission parameters (e.g., lymphocyte count, COPD duration), bypassing the need for complex algorithms or advanced laboratory tests. SHAP analysis further enhanced interpretability by revealing that low lymphocyte count was the strongest predictor of T2RF risk, likely reflecting infection-driven lymphopenia and associated immunosuppression (39–41). Prolonged COPD duration emerged as the second most influential factor, consistent with the progressive nature of airway remodeling, respiratory muscle dysfunction, and cumulative ventilatory impairment. Elevated RDW-CV, advanced age, and low eosinophil count also contributed independently, aligning with established evidence that chronic systemic inflammation, aging-related physiological decline, and altered immune responses modulate exacerbation severity and respiratory failure risk (37, 42).

Several limitations warrant consideration. First, the retrospective design inherently carries risks of selection and information bias, though stringent inclusion and exclusion criteria and standardized data collection protocols were implemented to minimize these effects. Second, the geographic restriction of our dataset to regional healthcare institutions in China raises concerns about population representativeness. Future validation should incorporate multicenter cohorts encompassing diverse demographic and socioeconomic populations to confirm generalizability. Third, the current model relies solely on admission parameters and excludes dynamic physiological variables such as serial arterial blood gas measurements or continuous respiratory monitoring, which could potentially enhance temporal sensitivity. Subsequent iterations could benefit from integrating real-time data streams from emerging wearable technologies. Finally, while our ensemble models achieved superior predictive performance, clinicians should exercise caution in their application. We strongly recommend employing this tool as part of a multimodal decision-making framework that synergizes computational predictions with bedside assessment and individualized risk–benefit analysis, particularly when managing patients with complex comorbidities.

Three critical pathways emerge for advancing this research frontier. Primarily, prospective multinational trials should be implemented through collaborative consortia to establish clinical validity across heterogeneous populations. Such initiatives would enable rigorous evaluation of model stability under varying healthcare infrastructures, particularly addressing disparities in resource-limited settings through adaptive validation protocols. Secondly, expanding the predictive framework through multi-modal data integration represents a crucial innovation direction. Incorporating dynamic biomarkers (e.g., serial lymphocyte counts, C-reactive protein trajectories), quantitative imaging biomarkers (e.g., CT-based airway quantification), and continuous physiological monitoring via medical Internet-of-Things devices could enable computational phenotyping of exacerbation subtypes. This systems medicine approach would facilitate mechanistic risk stratification while addressing current limitations in temporal resolution. Finally, translational implementation science must bridge the gap between algorithmic performance and clinical utility. Priority should be given to developing software-as-medical-device platforms featuring real-time analytics dashboards that interoperate with hospital electronic health record systems. Parallel development of clinician training programs and patient-facing predictive applications could foster a hybrid decision-making framework that synergizes artificial intelligence with human expertise. These synergistic efforts will ultimately drive precision exacerbation management through dynamic risk prediction, potentially modifying the natural history of COPD progression.

## 5 Conclusion

This study developed and externally validated a multimodal machine learning framework for the early prediction of in-hospital type 2 respiratory failure (T2RF) in patients with acute exacerbation of chronic obstructive pulmonary disease (AECOPD). Through a hybrid LASSO–XGBoost feature selection strategy, seven routinely available admission variables—lymphocyte count, eosinophil count, COPD duration, red cell distribution width–coefficient of variation (RDW-CV), age, hypertension, and sex—were identified as key predictors. The optimized XGBoost model achieved an area under the receiver operating characteristic curve (AUROC) of 0.699 (95% CI: 0.661–0.738) in the temporal–geographic external validation cohort, with 45.9% sensitivity and 79.0% specificity, while LightGBM demonstrated comparable discrimination (AUROC 0.700). SHAP analysis revealed that low lymphocyte count and prolonged COPD duration were the dominant risk drivers. A logistic regression-derived nomogram provided a clinically interpretable bedside tool with an external AUROC of 0.666. By restricting predictors to admission blood counts and demographics while deliberately excluding pulmonary ventilatory function, this framework enables immediate risk stratification at the point of care, even in settings where spirometry is unavailable. The externally validated models and the nomogram offer practical decision-support for frontline clinicians to identify high-risk patients and initiate timely interventions such as non-invasive ventilation. Nevertheless, the retrospective design and regional data sources warrant cautious interpretation. Future studies should prioritize prospective multicenter validation, integrate dynamic physiological monitoring, and embed the model into clinical decision support systems to bridge the gap between predictive analytics and bedside practice, ultimately reducing the mortality and morbidity burden of T2RF in AECOPD populations.

## Data Availability

The raw data supporting the conclusions of this article will be made available by the authors, without undue reservation.
