# Supplementary material for: Multimodal machine learning predicts type 2 respiratory failure in COPD exacerbations: a multicenter XGBoost model with clinical nomogram
Source: Front Med (Lausanne). 2026 Jul 15;13:1806614. doi: 10.3389/fmed.2026.1806614 (PMC13415698; doi:10.3389/fmed.2026.1806614)
Supplement: Supplementary file 1 [file Table_1.docx]

Supplementary Table S1: Missing value distributions by cohort

| Distribution s of missing value, n (%) | Training(4867) | Internal Test(2087) | External Validation(1252) |
| --- | --- | --- | --- |
| Age | 0 (0) | 0 (0) | 0 (0) |
| Sex | 0 (0) | 0 (0) | 0 (0) |
| Hypertension [Yes] | 0 (0) | 0 (0) | 0 (0) |
| Diabetes [Yes] | 0 (0) | 0 (0) | 0 (0) |
| CHD [Yes] | 0 (0) | 0 (0) | 0 (0) |
| COPD.duration | 698 (14.3%) | 294 (14.1%) | 118 (9.4%) |
| Smoke | 10 (0.2%) | 3 (0.1%) | 94 (7.5%) |
| PCT [μg/ml] | 1348 (27.7%) | 566(27.1%) | 304(24.3%) |
| D-D [mg/ml] | 428(8.8%) | 194(9.3%) | 134(10.7%) |
| NEUT# [×109/L] | 172 (3.5%) | 97(4.6%) | 197 (15.7%) |
| NEUT% [%] | 172 (3.5%) | 97(4.6%) | 53(4.2%) |
| EO# [×109/L] | 172 (3.5%) | 97(4.6%) | 302 (24.2%) |
| EO% [%] | 172 (3.5%) | 97(4.6%) | 164(13.1%) |
| LYMPH# [×109/L] | 172 (3.5%) | 97(4.6%) | 198 (15.8%) |
| LYMPH% [%] | 172 (3.5%) | 97(4.6%) | 53(4.2%) |
| NLR | 172 (3.5%) | 97(4.6%) | 198 (15.8%) |
| WBC [×109/L] | 172 (3.5%) | 97(4.6%) | 53(4.2%) |
| RDW-CV [%] | 172 (3.5%) | 97(4.6%) | 80(6.4%) |

Supplementary Table S2: Multicollinearity assessment and coefficient estimates of candidate predictors

| **Variable** | **Coefficient** | **VIF** |
| --- | --- | --- |
| Sex | -0.452 | 1.659 |
| Hypertension | -0.269 | 1.062 |
| EO# [×10^9^/L] | -0.255 | 5.729 |
| Smoke | 0.130 | 1.647 |
| RDW_CV | 0.109 | 1.021 |
| NEUT# [×10^9^/L] | -0.074 | 2.133 |
| LYMPH% [%] | -0.067 | 1.923 |
| Diabetes | -0.032 | 1.049 |
| Age | -0.031 | 1.083 |
| COPD.duration | 0.030 | 1.045 |
| EO% [%] | -0.015 | 6.223 |
| PCT | -0.014 | 1.052 |
| NLR | 0.006 | 1.935 |
